# Effects of electroconvulsive therapy on inflammatory markers and depressive symptoms in adolescents with major depressive disorder

**DOI:** 10.3389/fpsyt.2024.1447839

**Published:** 2024-10-25

**Authors:** Ning Du, Yvna Wang, Dandan Geng, Huan Chen, Fengming Chen, Li Kuang, Jiamei Guo

**Affiliations:** ^1^ Center for Mental Health, University-Town Hospital of Chongqing Medical University, Chongqing, China; ^2^ Department of Psychiatry, First Affiliated Hospital of Chongqing Medical University, Chongqing, China; ^3^ Dermatology and Cosmetic Center, University-Town Hospital of Chongqing Medical University, Chongqing, China; ^4^ Sleep Medicine Center, Shiyan Hospital of Traditional Chinese Medicine, Shiyan, Hu Bei, China

**Keywords:** adolescents, electroconvulsive therapy, major depressive disorder, inflammatory markers, HAMD-17

## Abstract

**Objective:**

Limited research exists on the use of electroconvulsive therapy (ECT) for adolescents with major depressive disorder (MDD). This study investigates the effects of ECT on inflammatory markers in adolescents aged 13-18 suffering from severe MDD, evaluating its efficacy in modulating cellular inflammatory markers and ameliorating depressive symptoms.

**Methods:**

A cohort of 38 adolescents with severe MDD received standard antidepressant therapy along with 6-8 ECT sessions spanning two weeks. A control group of 29 age-matched, healthy individuals was also assessed for comparative purposes. The investigation measured variations in depressive symptomatology and inflammatory marker levels (IL-1β, IL-6, IL-10) pre- and post-intervention.

**Results:**

Post-ECT, a substantial decrease in pro-inflammatory cytokines (IL-1β and IL-6) and an increase in the anti-inflammatory cytokine (IL-10) were noted. Participants who responded to the treatment showed a significant decline in HAMD-17 scores, which accentuates ECT’s therapeutic potential. Comparative analysis indicated a significant correlation between post-treatment inflammatory marker alterations and clinical improvement, implying that shifts in inflammatory state might serve as predictors of treatment response. Moreover, the mitigation of depressive symptoms exhibited a moderate correlation with post-treatment decrements in IL-1β and IL-6 levels, underscoring MDD’s intricacy and ECT’s comprehensive impact.

**Conclusion:**

While initial inflammatory marker levels did not predict the response to ECT, the post-treatment measures appeared to be linked to clinical improvement. These findings suggest ECT’s potential effectiveness in treating severe MDD in adolescents and point to the possible predictive value of inflammatory markers in therapeutic outcomes. The study contributes to our understanding of the biopsychosocial framework of MDD and indicates that ECT may be a viable treatment option for this population.

## 1 Introduction

Major depressive disorder (MDD) continues to pose a substantial challenge to adolescent mental health, leading to significant morbidity and functional impairment (1). Recent studies report an upward trend in the incidence of depression and suicide rates among Chinese adolescents (2, 3). While pharmacological interventions offer some benefits, they fail to achieve desired outcomes in all patients, calling for alternative treatment modalities. Electroconvulsive therapy represents such an alternative. As a psychiatric intervention, ECT uses electrical stimulation to induce epileptic seizures in anesthetized patients, with the goal of mitigating severe mental illnesses (4). Despite being regarded as the most efficacious treatment for severe depression (5), the underlying mechanisms of ECT remain largely elusive.

In studies on adult patients, inflammation has become recognized as a key factor in the neurobiology of depression. Elevated levels of pro-inflammatory cytokines, such as interleukin-6 (IL-6) and interleukin-1β (IL-1β), have been observed in patients with MDD (6–8). These cytokines are not only associated with the onset of the disease but also correlate with its severity and progression (9). Research showing that MDD patients have higher baseline levels of inflammatory cytokines compared to healthy controls highlights the potential therapeutic value of targeting these markers for treatment (10–13). Oxidative stress plays a crucial role in the pathophysiology of depression. It not only directly damages the nervous system but also influences neurotransmitter function, activates the immune system, and triggers inflammatory responses, contributing to the development and progression of depression through multiple pathways (14). While ECT’s effectiveness in adult depression is documented, its impact on adolescent inflammation is not yet fully understood (15–17). Adolescence, a period of significant biological and psychological evolution, may influence depression’s manifestation and treatment responsiveness. During which teenagers are more susceptible to inflammatory responses. Hormonal fluctuations during this stage modulate the immune system, making it more sensitive to inflammatory signals. Additionally, stressors such as academic pressure and interpersonal challenges can activate the immune system, leading to the release of pro-inflammatory cytokines, which are linked to the severity of depressive symptoms. Insights into how ECT modulates inflammatory pathways in adolescents could elucidate its therapeutic mechanisms and facilitate more precise interventions. Historical discourse on ECT has been marked by controversy, due to concerns over cognitive side effects and ethical issues, particularly in the adolescent population (18, 19). Despite these concerns, ECT is indispensable for managing treatment-resistant depression, noted for its capacity to rapidly ameliorate symptoms (20). However, research on ECT’s application in adolescents, especially its influence on inflammatory markers linked to depression’s pathophysiology, is limited.

Our study’s objective is to evaluate the impact of ECT on cellular inflammatory markers and depressive symptoms in adolescents. Involving 38 adolescents, the study administered ECT alongside antidepressants, with outcomes measured against those of a normative, age-matched control group. Through rigorous assessment of depressive symptoms and inflammatory markers pre- and post-ECT, we seek to illuminate the reciprocal relationship between inflammation and depression in this young population.

## 2 Method

### 2.1 Clinical characteristics

This investigation enrolled 52 inpatients from the First Affiliated Hospital of Chongqing Medical University during April to July 2023. Eligibility for participation was determined based on the following criteria: 1. A diagnosis of depression by psychiatric specialists in accordance with the Diagnostic and Statistical Manual of Mental Disorders (DSM-IV-TR); 2. A score of 17 or above on the Hamilton Depression Rating Scale; 3. No concurrent psychiatric disorders; 4. Absence of systemic illnesses, including diabetes, hypertension, hyperthyroidism, or hypothyroidism; 5. Age range from 13 to 18 years. The exclusion criteria at the commencement of the study were designed to eliminate patients with the following conditions: 1. comorbid physical conditions affecting mood; 2. immune or endocrine system disorders; 3. acute or recent infections or contagious diseases; 4. chronic physical illnesses or chronic inflammation; 5. use of anti-inflammatory drugs, immunomodulators, or hormonal treatments within one month prior to the study; 6. a self-reported history of epilepsy; 7. uncontrollable physical illness; 8. development of a physical illness during the study or decision to discontinue ECT treatment; and 9. refusal to sign the informed consent form. Within the ECT cohort, 14 patients ceased treatment due to reasons such as skin allergies (1 patient), refusal due to headaches (3 patients), and objection to recommended discharge (4 patients). Additionally, six individuals opted out of the ECT follow-up for personal reasons. A control group was established, consisting of volunteers aligned with the MDD patients by gender, age, and educational attainment. This group reported no lifetime mental disorders among immediate family members or a familial history of psychiatric conditions. Thus, the study’s conclusive participant makeup included 38 individuals in the ECT cohort and 32 in the control group. The research protocol received approval from the Ethics Committee of Chongqing Medical University(K2023-676). Informed consent was acquired in writing from legal guardians and all study participants.

### 2.2 Depression severity scores

The Hamilton Depression Rating Scale (HAMD-17) (21) is utilized for evaluating symptoms in depression patients, comprising 17 items. A higher aggregate score signifies a more pronounced level of depression, establishing the HAMD-17 as the clinical gold standard for gauging depressive disorder severity. Individuals experiencing a 50% or greater reduction in their HAMD-17 scores are identified as responders, whereas those who do not achieve this threshold are designated non-responders (22).

### 2.3 Electroconvulsive therapy

Before initiating ECT, each participant received comprehensive information about the therapy’s advantages and possible side effects, followed by the collection of their written informed consent. ECT treatments were administered from 8:00 AM to 12:00 PM at the First Affiliated Hospital of Chongqing Medical University, employing the Thymatron DGx system (Somatics, LLC, Lake Bluff, IL, USA) with electrodes positioned bilaterally on the temples (23). The protocol used a low pulse width setting of 0.25 and a current of 0.9A, with the initial energy typically adjusted to 50% of the participant’s age. Anesthesia and muscle relaxation were facilitated by propofol (1.5-2 mg/kg) and succinylcholine (0.5-1 mg/kg), respectively. Post-ECT, individuals were relocated to a recovery area for close monitoring of potential side effects, including dizziness, headaches, drooling, nausea, and vomiting. The ECT regimen entailed daily treatments for the initial 3-4 days, succeeded by sessions every alternate day, incorporating a two-day break during the weekend, amounting to 6-8 sessions within two weeks. Concurrently, participants maintained their prescribed regimen of antidepressants throughout the ECT treatment phase.

### 2.4 Blood samples

Blood samples were obtained in the morning after a 12-hour fasting period and while participants were in a supine position at both the study’s inception and its conclusion four weeks later. A 5 ml venous blood sample from each patient was collected on ice using EDTA as the anticoagulant. Within 30 minutes of collection, the samples were centrifuged at 1000g and a temperature of 2-8°C for 15 minutes. This was followed by an additional centrifugation step of the separated plasma at 10000g for 10 minutes at 2-8°C, ensuring the complete elimination of platelets. Levels of IL-1β, IL-6, and IL-10 were quantified through enzyme-linked immunosorbent assay (ELISA) using commercial kits, in accordance with the manufacturer’s guidelines [eBioscience Inc (San Diego, CA, USA) for IL-6, and R&D Systems Europe Ltd. (Abingdon, UK) for IL-1β and IL-10].

### 2.5 Statistical analyses

Continuous variables conforming to a normal distribution were analyzed using independent t-tests for statistical comparisons. The Mann-Whitney U test was applied to continuous variables that did not follow a normal distribution. Categorical data, expressed as frequencies and percentages, were examined for statistical differences using either Pearson’s chi-square test or Fisher’s exact test, depending on the data’s distribution. Multiple comparisons were corrected for significance using the Bonferroni method. Logistic regression analysis was used to evaluate the predictive effects of multiple variables on the outcomes of therapy. The association between variables was explored through Pearson’s correlation test or Spearman’s rank correlation test, based on the normality of the data distribution. A P-value below 0.05 was deemed to indicate statistical significance. The analyses were conducted using IBM SPSS Statistics software, Version 26 for Windows.

## 3 Results

### 3.1 Demographics and baseline characteristics

A comparative analysis was conducted between the control and ECT groups, examining variables such as age, gender, and years of education, along with baseline levels of IL-1β, IL-6, and IL-10. No significant disparities were identified in age, gender, and educational attainment across the groups (p > 0.05). However, notable differences in baseline levels of inflammatory markers—IL-1β, IL-6, and IL-10—were evident between the groups (p < 0.001), as denoted by an asterisk. This indicates that the ECT group exhibited significantly elevated baseline levels of IL-1β and IL-6, whereas IL-10 levels were considerably lower compared to the control group (**Table 1**).

**Table 1:** Baseline characteristics and inflammatory biomarker levels in control and ECT groups.

Characteristic	Control(n=29)	ECT(n=38)	F	t	P-value
Age,years,(median,IQR)	15(14.5-16.5)	15(14-16)	1.42	-0.77	0.34
Female,n(%)	17(53.8%)	31(81.6%)	7.98	1.76	0.72
Years of schooling,(median,IQR)	8(7.5-9)	8(7-9)	3.77	-0.57	0.49
Baseline IL-1β,pg/ml,(mean ± SD)	43.03 ± 9.85	83.92 ± 16.49	3.37	-8.41	<0.001*
Baseline IL-6,pg/ml,(median,IQR)	28.55(22.26)	55.85(43.28-32.07)	10.36	-8.03	<0.001*
Baseline IL-10,pg/ml,(mean ± SD)	1099.96 ± 162.41	447.82 ± 107.81	3.49	16.44	<0.001*

ECT, electroconvulsive therapy; IL, interleukin; IQR, Interquartile Range; SD, Standard Deviation,*P<0.05.

### 3.2 Changes in IL-1β, IL-6, IL-10 levels, and HAMD-17 scores following ECT

**Figure 1:**
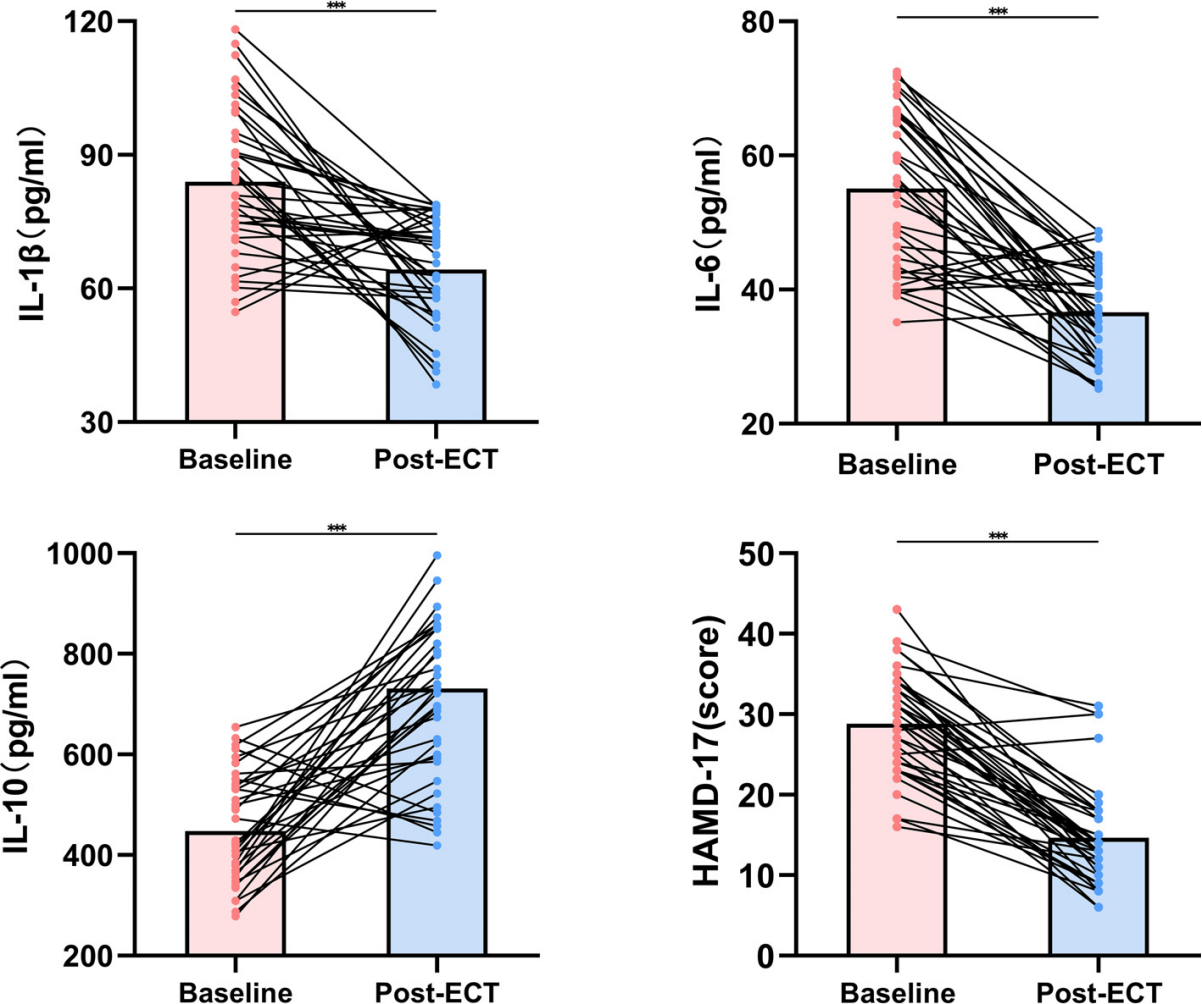
Changes in IL-1β, IL-6, IL-10 levels, and HAMD-17 scores following ECT. ***mean P<0.001.

Subsequent to ECT treatment, significant modulation was noted in levels of both pro-inflammatory and anti-inflammatory biomarkers. The mean levels of IL-1β and IL-6 markedly decreased post-ECT (p<0.001), implying a potential anti-inflammatory action of the therapy. In contrast, IL-10 levels experienced an increase after ECT, supporting the anti-inflammatory hypothesis underlying ECT’s mechanism (p<0.001). These alterations suggest a cytokine profile shift towards an anti-inflammatory state following ECT. Aligning with immunological evidence, the severity of depressive symptoms, as gauged by HAMD-17 scores, significantly diminished after the intervention, reflecting a positive clinical response (p<0.001). The degree of symptom reduction underscores ECT’s effectiveness in mitigating the clinical symptoms of severe depression among adolescents (**Figure 1**).

### 3.3 Comparative analysis of demographic statistics, baseline and post-treatment levels of inflammatory markers, and HAMD-17 scores between ECT non-responders and responders.

The study analyzed 38 participants, divided into ECT non-responders (n=14) and responders (n=24), based on a significant reduction in HAMD-17 scores. Demographic analysis showed a median age of 15 years with no substantial difference between non-responders and responders (p=0.865). The cohort was predominantly female, comprising 81.6% of participants, with no significant gender disparity observed between the groups (p=0.615). Education levels, quantified by years of schooling, were similar across groups, with a median value of 8 years. The frequency of ECT sessions was consistent across groups (p=0.307), suggesting a standardized treatment regimen.

At the study’s inception, no significant differences were detected between the two groups in terms of IL-1β, IL-6, IL-10 levels, or baseline HAMD-17 scores. However, the post-treatment results varied. After ECT, Bonferroni correction revealed a significant reduction in the median IL-1β levels in the responder group compared to the non-responder group (p=0.006). Additionally, the mean IL-6 levels post-treatment were significantly lower in the responder group than in the non-responder group (p<0.001). Conversely, the IL-10 levels in the responder group showed a significant increase compared to the non-responder group (p=0.011). Notably, depressive symptoms improved following ECT, with the median HAMD-17 score in the responder group decreasing to 11.5, a statistically significant change compared to the non-responder group (p<0.001) (**Table 2**).

**Table 2:** Comparative analysis of demographics, treatment sessions, baseline and post-treatment biomarker levels, and HAMD-17 scores between non-responders and responders to ECT.

Variables	Total(n=38)	Non-responder(n=14)	Responder(n=24)	F	t/χ2	P-value
Age,years,(median,IQR)	15(14-16)	14.5(14-16.25)	15(14-16)	1.00	0.08	0.87
Female,n(%)	31(81.6%)	12(85.7%)	19(79.2)	1.03	12,26	0.62
Years of schooling,(median,IQR)	8(7-9)	7.5(7-9.25)	8(7-9)	1.00	0.08	0.86
Number of ECT sessions,n,(median,IQR)	6(6-7)	6.5(6-7.25)	6(6-7)	0.50	-0.92	0.31
Medication						
Sertraline,n(%)	18(47.4%)	5(35.7%)	13(54.2%)	1.46	0.11	0.27
Fluoxetine,n(%)	21(55.3%)	10(71.4%)	11(45.8%)	4.39	0.42	0.13
Venlafaxine,n(%)	9(23.7%)	2(14.3%)	7(29.2%)	5.20	10.53	0.30
Escitalopram,n(%)	2(5.3%)	1(7.1%)	1(4.2%)	0.60	30.42	0.69
Quetiapine,n(%)	9(23.7%)	3(21.4%)	6(25%)	0.25	10.53	0.80
Olanzapine,n(%)	5(13.2%)	2(14.6%)	3(12.5%)	0.09	20.63	0.87
Aripiprazole,n(%)	2(5.3%)	1(7.1%)	1(4.2%)	0.60	30.42	0.69
Risperidone,n(%)	3(7.9%)	2(14.3%)	1(4.2%)	5.16	26.95	0.26
Tandospirone,n(%)	3(7.9%)	1(7.1%)	2(8.3%)	0.07	26.95	0.89
Baseline IL-1β,pg/ml,(mean ± SD)	83.92 ± 16.49	83.93 ± 16.49	64.21 ± 12.15	1.01	1.74	0.09
Baseline IL-6,pg/ml,(median,IQR)	55.85(43.27-66.41)	55.01(42.53-66.06)	56.18(44.18-66.65)	0.07	0.22	0.83
Baseline IL-10,pg/ml,(mean ± SD)	447.82 ± 107.81	447.82 ± 107.82	730.67 ± 181.78	1.29	1.56	0.13
Baseline HAMD-17,score,(mean ± SD)	27.82 ± 6.42	27.14 ± 6.80	28.21 ± 6.311	9.27	0.89	0.64
Post-IL-1β,pg/ml,(median,IQR)	66.58(54.12-75.09)	72.73(67.90-77.14)	59.90(53.25-71.85)	1.01	2.39	0.03*
Post-IL-6,pg/ml,(mean ± SD)	36.62 ± 6.98	40.43 ± 4.05	34.39 ± 7.42	5.40	3.24	0.01*
Post-IL-10,pg/ml,(mean ± SD)	730.67 ± 181.77	655.80 ± 156.28	774.35 ± 184.25	0.74	-2.11	0.04*
Post-HAMD-17,score,(median,IQR)	13(10-18)	18(14.5-27.75)	11.5(9-14)	0.74	4.17	<0.01*

ECT, electroconvulsive therapy; IL, interleukin; SD, Standard Deviation; IQR, Interquartile Range; HAMD-17, 17-item Hamilton Depression Scale, *P<0.05.

### 3.4 Binary logistic regression analysis for ECT response

The analysis revealed that neither age (p=0.216) nor gender was a significant predictor of the response to ECT. Similarly, the number of ECT sessions did not significantly correlate with treatment outcomes (p=0.443). Furthermore, the baseline severity of depression, as assessed by the HAMD-17, did not significantly forecast the ECT response (p=0.543).

An intriguing discovery was made regarding post-treatment levels of inflammatory biomarkers. Specifically, lower levels of IL-1β post-treatment were significantly linked to a favorable response to ECT (p=0.041), implying that the antidepressant effects of ECT could be partially attributed to its anti-inflammatory properties. A comparable trend was noted with IL-6, where decreased levels post-treatment were associated with improved therapeutic outcomes (p=0.038). While post-treatment IL-10 levels did not achieve statistical significance, they showed a positive trend towards correlating with a successful ECT response (p=0.080) (**Table 3**).

**Table 3:** Binary logistic regression analysis for ECT response.

Variable	B	SE	Wald	P-value	OR	95%CI
Age	-1.26	1.01	1.53	0.22	0.29	0.039-2.086
Female	0.73	2.17	0.11	0.74	2.08	0.029-148.628
Number of ECT sessions	0.65	0.85	0.59	0.44	1.93	0.361-10.271
Baseline HAMD-17	0.07	0.11	0.37	0.54	1.07	0.863-1.324
Post-IL-1β	-0.33	0.16	4.18	0.04*	0.72	0.520-0.986
Post-IL-6	-0.79	0.38	4.29	0.04*	0.46	0.217-0.959
Post-IL-10	0.03	0.02	3.07	0.08	1.03	0.997-1.059

SE, Standard Error; OR, Odds Ratio; CL, Confidence Level; ECT, electroconvulsive therapy; HAMD-17, 17-item Hamilton Depression Scale; IL, interleukin. *P<0.05.

### 3.5 Correlation between changes in depression severity and inflammatory biomarkers

**Figure 2:**
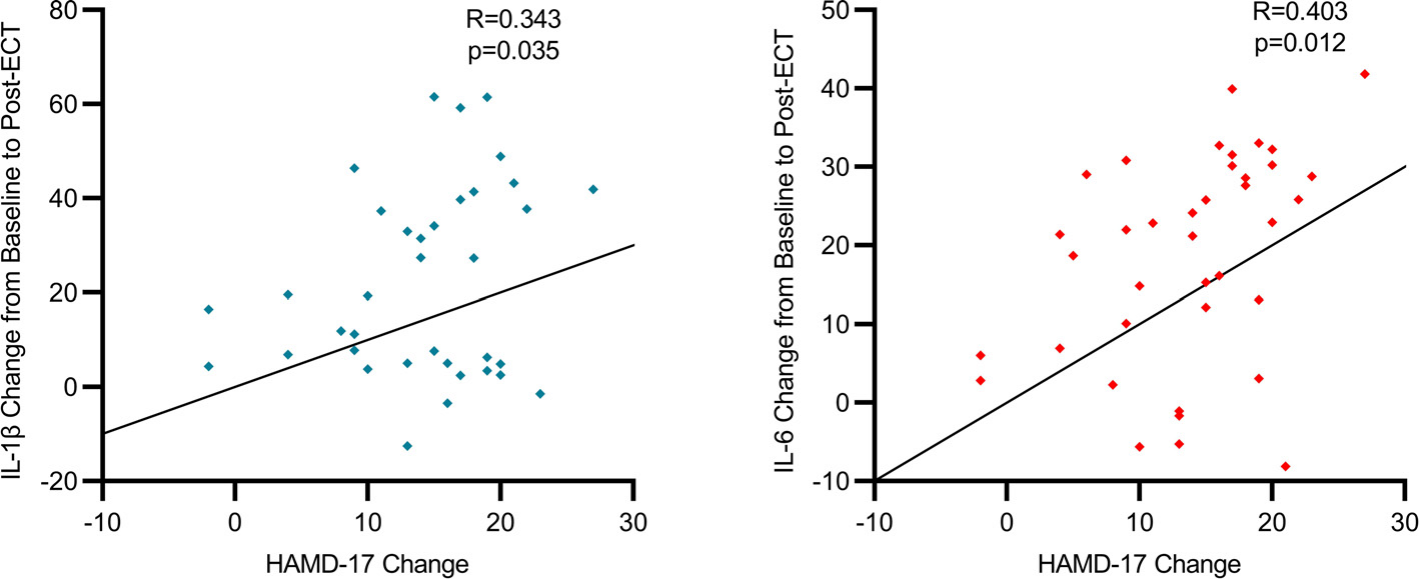
Correlation between changes in depression severity and inflammatory biomarkers.

Our study has uncovered a notable correlation between the severity of depressive symptoms in individuals with severe depression and the levels of specific inflammatory biomarkers before and after ECT. We observed a moderate positive correlation between the variations in HAMD-17 scores and IL-6 levels (R=0.403, p=0.012), suggesting a tendency for IL-6 levels to decrease as depressive symptoms improve. This pattern was uniformly observed across the participant group, clearly illustrating that a decline in IL-6 levels is associated with clinical improvements in depressive symptoms. Additionally, alterations in HAMD-17 scores exhibited a moderate positive correlation with IL-1β levels (R=0.343, p=0.035), indicating that the reduction in depressive symptoms post-ECT is linked to decreased IL-1β levels. These results imply that ECT not only has the capability to diminish inflammation but also suggests that the degree of inflammation reduction might correlate with the amelioration of depressive symptoms, reflecting a bidirectional relationship. Scatterplots visually depict the trajectories of individual patients, predominantly showing a concurrent reduction in both HAMD-17 scores and inflammatory biomarkers following ECT (**Figure 2**).

## 4 Discussion

In this study, we explored the potential effects of electroconvulsive therapy on inflammatory markers in adolescents with major depressive disorder and examined its possible efficacy in alleviating depressive symptoms. By analyzing 38 adolescent patients undergoing ECT, we observed that ECT was associated with improvements in clinical depressive symptoms and changes in inflammatory marker levels.

At baseline, there were no significant demographic differences between the control and ECT groups, suggesting that the observed effects on inflammatory biomarkers and depressive symptoms could potentially be attributed to the ECT intervention. Notably, the ECT group exhibited higher levels of pro-inflammatory biomarkers (IL-1β and IL-6) and lower levels of the anti-inflammatory biomarker (IL-10) at baseline. This imbalance may support the hypothesis that a pro-inflammatory state could be involved in the pathophysiology of adolescent depression, and that ECT might exert therapeutic effects by helping to restore immune balance. This observation is consistent with previous findings that suggest an imbalance of inflammatory markers may be associated with depression’s pathophysiology (24). For example, Miller et al. emphasized the potential role of elevated pro-inflammatory cytokine levels in the development of MDD (2). In our study, after ECT treatment, levels of pro-inflammatory cytokines appeared to decrease, while levels of anti-inflammatory cytokines increased, which could indicate that ECT modulates inflammatory responses. However, it is important to note that these findings align with Kranaster et al., who reported antidepressant effects associated with reduced innate immune activity in cerebrospinal fluid, but other studies present a more nuanced picture. For instance, van Buel et al. found that ECT did not prevent lipopolysaccharide-induced microglial activation, suggesting that the impact of ECT on inflammatory pathways may vary depending on specific conditions or immune challenges (25). Additionally, Jansson et al. reported that repeated ECT increased the number of vessel-associated macrophages in the rat hippocampus, highlighting the complex and potentially varied effects of ECT on the brain’s immune environment (26).

Our study found that ECT was associated with a reduction in HAMD-17 scores, which appeared to correlate with changes in inflammatory marker levels. Specifically, levels of pro-inflammatory cytokines IL-1β and IL-6 showed a tendency to decrease, while levels of the anti-inflammatory cytokine IL-10 increased. These findings are in line with Järventausta et al., who suggested that changes in IL-6 levels during ECT might reflect therapeutic response (27). Additionally, Wennström et al. demonstrated that ECT induced the proliferation of NG2-expressing glial cells in the adult rat hippocampus, which could potentially explain the observed trends in IL-10 levels and improvement in depressive symptoms in our study (28). This cellular-level change supports the hypothesis that ECT may exert its therapeutic effects by promoting cellular regeneration and repair in certain brain regions. However, more research is needed to fully confirm these relationships. Our findings also suggested a potential association between inflammation and depressive symptoms, indicating that changes in inflammatory marker levels might serve as indicators for predicting ECT treatment outcomes (29). The changes in inflammatory markers, rather than baseline levels, appeared to be associated with clinical improvement, which is consistent with the trends observed in Dowlati et al.’s meta-analysis linking changes in inflammatory markers to treatment outcomes in depression (30). This has important implications for personalized treatment, potentially aiding clinicians in adjusting therapeutic strategies for better outcomes.

We further explored the correlation between the severity of depressive symptoms and inflammatory biomarkers, finding a moderate positive correlation between changes in HAMD-17 scores and levels of IL-6 and IL-1β. This suggests a potential link between treatment effects on both depression and inflammation. This bidirectional relationship highlights the complexity of MDD and suggests that ECT may play a role not only as a neuromodulatory intervention but also in modulating inflammatory responses. The reduction in IL-6 and IL-1β levels was closely linked to improvements in depressive symptoms, supported by Rush et al.’s findings that ECT can modulate immune markers and influence treatment response. Studies have also shown that ECT may regulate the inflammatory state by affecting specific immune cells, such as macrophages (31). Roman et al. found that chronic ECT could modulate macrophage immune function, potentially explaining the changes in pro- and anti-inflammatory factor levels observed in our study (32, 33).

For adult patients, particularly younger individuals with higher metabolic activity, the acute inflammatory response following ECT is typically more pronounced, characterized by a rapid increase in pro-inflammatory cytokines such as IL-1 and IL-6 in the short term (27). This phenomenon is more evident in younger patients, likely due to the heightened activity and responsiveness of their immune systems. However, as treatment progresses, the levels of pro-inflammatory cytokines gradually return to baseline, indicating that the immune response in younger patients stabilizes following ECT (34).

This study has several limitations. First, the small sample size limits the statistical power and reduces the generalizability of the findings, necessitating validation through larger-scale studies in the future. Second, the absence of a control group not receiving ECT hinders the ability to accurately assess the direct impact of ECT on changes in inflammatory markers, as the results may be confounded by the natural course of the disease or other concurrent treatments. Lastly, participants were on different psychopharmacological regimens, and these medications may have independently influenced inflammatory markers.

In summary, our findings suggest that ECT may be effective in improving inflammatory marker levels and depressive symptoms in adolescents with major depressive disorder. These results provide some insights into the treatment of depression and offer potential directions for future research, particularly in exploring the mechanisms by which ECT might modulate inflammatory pathways. Additionally, further investigation is needed to determine how these biomarkers could be used to optimize treatment protocols. Further randomized controlled trials are needed to validate our findings and enhance the quality and reliability of research in this field.

## Data Availability

The raw data supporting the conclusions of this article will be made available by the authors, without undue reservation.
